# Expression analysis of genes enriched in the pulvinus of *Lotus japonicus*

**DOI:** 10.5511/plantbiotechnology.24.1030a

**Published:** 2025-03-25

**Authors:** Akari Harada, Mako Onori, Moeka Ooki, Nobuyuki Kanzawa

**Affiliations:** 1 Department of Materials and Life Sciences, Faculty of Science and Technology, Sophia University

**Keywords:** auxin, leaf movement, legume, pulvinus, transcriptome

## Abstract

The pulvinus is a unique motor organ found in leguminous plants. The motor cells surrounding the central vascular bundle of the pulvinus are divided into extensor and flexor halves. The asymmetric change in turgor pressure of the motor cells of the extensor/flexor halves is the driving force behind nyctinastic leaf movement. Omics analysis has recently revealed genes involved in pulvinar development and function, but the molecular mechanism orchestrating the pulvinar movement remains elusive. In this study, we investigated genes predominantly and highly expressed in the pulvinus to find out key genes involved in the regulation of nyctinastic movement. Gene expression in both the pulvinus and stem at dawn and dusk was examined using RNA sequencing analysis. As a result, several genes were identified that preferentially change in expression in the pulvinus at dawn. Among the genes, we first focused on genes that are more highly expressed in the pulvinus than in the stem and validated the results by reverse transcription-polymerase chain reaction (RT-PCR). We further focused on auxin-related genes, as auxin was found to be preferentially expressed in the pulvinus and has been reported to be involved in the regulation of nyctinastic leaf movement. Quantitative real-time PCR and in situ hybridization analyses revealed that at least two auxin-related genes, *IAA19*/*FLS1*, are dominantly expressed in the pulvinus. Thus, we provided a new dataset to identify genes involved in the regulation of nyctinastic leaf movement.

## Introduction

Due to the sessile nature of plants, developmental plasticity and adaptability are essential for their survival as well as for habitat expansion. One of the adaptations to changes in the environment is the presence of a well-orchestrated endogenous circadian rhythm. The circadian rhythm repeats in a 24-hour cycle and involves many biological phenomena. In plants, events such as stomatal opening and closing, hypocotyl elongation, and flowering are controlled by circadian rhythms (Harmer 2009). The first report of circadian rhythms involved experiments using *Mimosa pudica*. Plants placed in continuous darkness maintained periodic leaf opening and closing movements even in the absence of light, indicating the existence of a mechanism in the plant to maintain diurnal rhythms (de Mairan 1729). Most legumes open their leaves during the day and close them at night by controlling their internal circadian clock. This leaf movement is known as nyctinasty, a unique and fascinating movement that has attracted the interest of scientists for centuries (Mano and Hasebe 2021; Satter and Galston 1981; Wang et al. 2023; Weintraub 1952). Nyctinasty is driven by the daily rhythmic expansion and contraction of motor cells located in the pulvinus at the base of a leaf or leaflet. This deformation in motor cells is regulated by the day/night cycle of osmotic pressure changes, which in turn are influenced by the influx and efflux of ions through ion channels embedded in the cell membrane. This intricate process illustrates the complex interplay between cellular mechanisms and environmental cues in nature (Satter et al. 1974). In *Lotus japonicus*, the motor cells are distributed in the extensor tissue (abaxial half) and flexor tissue (adaxial half) of the pulvinus. The imbalance between the efflux or influx of ions from the extensor and flexor motor cells, followed by the change in turgor pressure of the cells, causes a change in cell volume and consequent nyctinastic leaf movement (Satter and Galston 1981; Satter et al. 1992). This turgor-driven leaf movement is mechanistically similar to the stomatal opening and closing movement of guard cells (Satter et al. 1974). Mechanisms orchestrating stomatal movements have been studied in *Arabidopsis thaliana*, and detailed molecular mechanisms have been reported (Kinoshita et al. 2003; Shimazaki et al. 2007). However, it is not clear whether factors involved in the stomatal movement are also involved in nyctinastic movement at the pulvinus. In the model legume *Medicago truncatula*, genes involved in pulvinar development and function are studied by comparing gene expression between *plp* mutants with petiole-like pulvinus and wild-type plants (Zhou et al. 2012). They also report that two genes, DWARF4a, which is an ortholog of Arabidopsis DWARF4, and MIO1 (MINI ORGAN 1), which encodes an F-box protein, are involved in the regulation of pulvinus development in *M. truncatula* (Zhao et al. 2021; Zhou et al. 2021). In this study, we performed transcriptome analysis using RNA sequencing (RNA-seq) to identify genes involved in the regulation of nyctinastic movement of *Lotus japonicus*. In addition, since several auxin-related genes were found to be preferentially expressed in the pulvinus using RNA-seq results and have been reported to be involved in the regulation of nyctinastic leaf movement, we investigated the temporal expression changes and tissue distribution of three auxin-related genes. Auxin functions as a morphogen in plant tissue formation, synthesized in shoot apical tissues and transported to roots, creating a concentration gradient. The localization of PIN protein on the cell membrane is crucial for auxin’s polar transport (Vanneste and Friml 2009). Auxin signaling begins when auxin binds to TRANSPORT INHIBITOR RESPONSE1/AUXIN SIGNALING F-BOX (TIR1/AFB) protein, leading to the ubiquitination and degradation of the transcription repressor auxin/indole-3-acetic acid (Aux/IAA) (Gray et al. 2001; Tan et al. 2007). This leads to the release of auxin response factor (ARF), a transcription factor that bind to the auxin-responsive element (AuxRE) to regulate gene expression (Tiwari et al. 2003; Ulmasov et al. 1997). Recently, Bai and colleagues performed transcriptomic and proteomic analyses to identify genes involved in pulvinar formation and function by comparing the wild-type of *Medicago truncatula* and *plp* mutant. They found that auxin-related genes have pivotal roles in the development and formation of the pulvinus (Bai et al. 2022). In addition to its role in development, auxin has long been thought to be involved in diurnally regulated leaf movement (Satter et al. 1972; Watanabe and Umrath 1983). Auxin-induced IAA degradation results in the induction of small auxin up-regulated RNA (SAUR) expression in *Arabidopsis* stomatal cells, and SAUR is known to be involved in maintaining stomatal opening by maintaining the phosphorylation of H^+^-ATPase (Perez-Henriquez and Yang 2023; Wong et al. 2021). Thus, we investigated the expression and distribution of auxin-related genes in *L. japonicus* in this study. Our results provide a new dataset to characterize the molecular basis for understanding nyctinastic leaf movement.

## Materials and methods

### Plants

*L. japonicus* MG-20 ‘Miyakojima’ was used in this study. Plants were grown on shelves in the culture room conditioned at 25°C under LD (16 h light : 8 h dark; 16L : 8D) light periods at approximately 150 µE m^−2^ s^−1^. Leaves from the shoot tip to the third to fourth node were excised at zeitgeber time (ZT) points every four hours for diurnal expression analysis.

### Gene expression analysis by semiquantitative RT-PCR and qRT-PCR

To examine the tissue specificity of gene expression, total RNA was isolated from plant leaves, pulvini, and stems at ZT4 and ZT16 using ISOSPIN Plant RNA (NIPPON GENE, Tokyo, Japan) according to the manufacturer’s protocol, and complementary DNAs (cDNAs) were prepared using High-Capacity cDNA-Reverse Transcription Kits (Promega, Tokyo, Japan) with an oligo-dT primer and stored at −20°C until use. Primers designed to amplify the fragments to approximately 500 bp (Table 1) were used for 30 cycles of PCR. Semiquantitative analysis was performed by measuring band intensities of ethidium bromide-stained gels by densitometric analysis using ImageJ software (Schneider et al. 2012), and normalized to that of *PROTEIN PHOSPHATASE 2A* (*PP2A*). The data obtained were analyzed using R® statistical software (Ihaka and Gentleman 1996), and are presented as the mean±standard error of three biological replicates. Data were then evaluated statistically using a two-way ANOVA, followed by Tukey’s post hoc test to demonstrate the significance of between-group differences. Statistical significance was set at *p*<0.05.

**Table 1:** Table 1. Primers for RT-PCR.

Gene	Forward primer (5′-3′)	Reverse primer (5′-3′)	Product size (bp)	Gene ID
*SKOR*	AAGGCTTCTATCTAATGGGATG	GCGTTTCATGGACTAAATACAGCTT	503	Lj2g3v2541790
*SWEET*	CATCTATCTCAGTTACTGCCCCAA	CAGTTCCATGAACAGGCTTCTC	548	Lj4g3v2882250
*ALMT*	GACTCTTGCATCAGACTAGCTGA	CTCATCATCATCCTCTCTGAACTC	486	Lj5g3v2261760
*ZnfC2H2*	ATTGAACAAGGGCCAGTATTGG	CTGCTTCATCTTCTTCTTGCAAG	512	Lj3g3v0488270
*INT*	CCAGTCCAATGAATTGGCTCTTC	CTCTCCTTCCAAATCAGCTCCAC	519	Lj5g3v2300410
*NRT*	CTCCTCCAGAGAATTGGAATTG	CCCTTTGAACCTAACTCCACATC	509	Lj2g3v1349210
*FLS1*	GGATGGGAAGAAAGGTTGGGT	GAGGCCCAACTTCATGCTCT	545	Lj5g3v1865700
*CYP82C2*	AGTGAGTTATTTGAGCACATGACA	GTGCCCAGGGACATCACTAC	502	Lj3g3v0718770
*CML41*	AACGCAGAATTTGAGCGTGT	TGAACTCTTCAAAGCAAAGCAG	412	Lj3g3v0766150
*IAA19*	AGCTAAGGTTGGGTTTGCCT	GCGCCTTCTATGAATCCC	523	Lj3g3v2247090
*BAP2*	CAGCAAAAGCCACACACACA	CACCGCCAGTAACAGAACCT	503	Lj1g3v2609230
*PP2A*	AGTCATGGCGTGTCCGTT	CTATGATAGCAAGTCGAACTC	498	Lj2g3v0742070

For the quantitative real-time PCR (qRT-PCR), RNA was prepared from the pulvinus every 4 h. The primer pairs for *IAA19*, *FLS1*, and *ILL4* were designed to flank one intron, and the amplified fragments were approximately 100 bp (Table 2). qRT-PCR was performed on a QuantStudio™ 3 Real-Time PCR System (Thermo Fisher Scientific, Waltham, MA, USA) using Power SYBR Green PCR Master Mix (Applied Biosystems, Warrington, UK) following standard protocols. The starting quantity was estimated from critical thresholds using the standard curve method. Data for each sample were normalized to the respective *PP2A* expression level. The relative expression level of each gene was expressed as 2^−ΔΔCt^.

**Table 2:** Table 2. Primers for qRT-PCR of auxin-related genes.

Gene	Forward primer (5′-3′)	Reverse primer (5′-3′)	Product size (bp)	Gene ID
*IAA19*	TCCCTTGGGAGATGTTTACC	CCCTTTAAAGATCCCTTCTG	100	Lj3g3v2247090
*FLS1*	CAGGGTCTTCAGGCCTCTAG	TGCTCATTACCTCCATTTGG	100	Lj5g3v1865700
*ILL4*	TCCAGTTGCAGTTACAGGTG	CCACCATTTCCTGCATAGGA	104	Lj3g3v2718800
*PP2A*	CATTATTCCCCAGGTTTTGG	AATCTCTGAGCCCAGAACAG	106	Lj2g3v0742070

### Transcriptome analysis

Total RNAs were isolated from pulvini and stems at ZT4 and ZT16, using ISOSPIN Plant RNA as described above. RNA sequence library construction, sequencing, mapping, and expression analysis were performed by DNAFORM (Yokohama, Kanagawa, Japan). In brief, quality confirmation was conducted using a Bioanalyzer (Agilent Technologies, Palo Alto, CA, USA). RNA-seq libraries were sequenced using paired-end reads (50+25 bp) on a NextSeq instrument (Illumina Inc., San Diego, CA, USA). Obtained reads were mapped to the *Lotus* genome using STAR. Gene read counts were obtained from the mapping data using the feature Counts. The expression level of each gene was quantified by RSEM and pairwise comparisons were performed by DEseq2. FPKM (fragments per kilobase of exon per million fragments mapped) values normalized from the count data of each single biological experiment were used to compare expression levels between samples. Fold change (FC) was calculated with the FPKM value. MA plots were employed to visualize differential gene expression (DEG) (MA stands for log_2_ ratio (M) versus the log_2_ of the mean average (A)) (Anders and Huber 2010). Adjusted *p*-value (padj) <0.05 and |Fold Change| >2 were set as the filtering criteria for significant differential expression in the pulvinus (Supplementary Tables S1, S2 and S3). Gene ontology (GO) enrichment analysis (Supplementary Tables S4, S5 and S6) was performed with the GO program of Lotus Base (Mun et al. 2016). Obtained sequences were deposited in DDBJ DRA, accession number DRA013270.

### Tissue fixation

Compound leaves of *L. japonicus* are composed of two basal leaflets and distal leaflets, with one terminal and two lateral leaflets (Wang et al. 2013). Distal leaflets (trifoliate) were cut at the rachis and fixed in 4% paraformaldehyde (PFA) in 1 M phosphate buffer (PB) and allowed to stand at 4°C. After 24 h of fixation, most of the leaflet lobes were excised. Then, tissue fragments consisting of rachis, pulvinus, and a part of the leaflet were washed twice with phosphate-buffered saline (PBS) for 5 min each. The tissue fragments were further incubated in 0.5 M ethylenediaminetetraacetic acid (EDTA) at 4°C overnight, followed by gently shaking at 4°C for another night. After washing with PBS twice for 5 min each, the samples were kept in PBS at 4°C for 24 h. Tissue fragments were dehydrated by dipping into a graded ethanol series of 25, 50, and 75% ethanol in PBS, followed by 70, 80, 90, and 95% ethanol for 1 h each, and the samples were kept at −30°C in 99.5% ethanol.

### Tissue cryosection

Samples were rehydrated by dipping in 75, 50% ethanol, followed by 50, 25% ethanol in PBS for 30 min each. After washing with PBS twice for 5 min each, samples were immersed in 30% sucrose in PBS at room temperature for 1 h. Samples were transferred to fresh 30% sucrose in PBS and kept at 4°C overnight. After washing in Optimal Cutting Temperature (OCT) compound (Sakura Finetek, Tokyo, Japan) three times, the samples were embedded in OCT compound and kept at −80°C until use. Tissue sections (20 µm thick) were prepared from the embedded sample using a cryostat (NX50, ThermoFisher, Tokyo, Japan). The specimens were air-dried at room temperature for over 1 h and the OCT compound was washed out with distilled water.

### cDNA cloning and RNA probe synthesis

Among the genes listed in the RNAseq data, three genes related to auxin signaling that appear to be relatively highly expressed in the pulvinus were partially sequenced to identify their expression sites (Supplementary Table S7). Primers for *IAA19*, *FLS1*, and *ILL4* were designed to amplify a unique gene sequence by referring to the sequence provided by Lotus Base and summarized in Supplementary Table S8. Amplification was done using PrimeSTAR Max DNA polymerase (TaKaRa Bio, Shiga, Japan) and the amplified fragment was subcloned into a pGEM-T easy vector (Promega). Aliquots were used to transform competent cells of *Escherichia coli*, DH-5α. An isolated cDNA clone (Supplementary Figure S2) was sequenced and used as a template for RNA-probe synthesis.

The cDNA template for each gene was digested with an appropriate restriction enzyme to produce sense- and antisense-RNA probes using a DIG RNA Labeling Kit (SP6/T7) (Sigma-Aldrich, St. Louis, MO, USA). Probe synthesis was confirmed by gel-electrophoresis and the amount of probe used for in situ hybridization was determined by dot-blotting.

### In situ hybridization analysis

To prevent the sections from coming off the glass slides during the hybridization process, the sections were sprayed with 70% ethanol and dried completely at 37°C on an extension board. The same treatment was repeated several times and the sections were fixed again with 4% PFA for 15 min. After rinsing with PBS, the specimens were immersed in 0.2 M HCl for 10 min and washed with PBS. Specimens were immersed in 0.1 M triethanolamine (TEA) pH 8.0 for 1 min and 0.1 M TEA containing 0.25% acetic anhydride for 10 min, followed by rinsing with PBS. Specimens were dehydrated by serially immersing in 70, 80, 90, and 99.5% ethanol for 15 s each and dried.

The hybridization buffer (HB) contained 50% formamide, 5 × SSC (Wako Pure Chemical, Osaka, Japan), 0.1% Tween-20, 50 µg ml^−1^ tRNA, and 50 µg ml^−1^ heparin. After preheating at 85°C for 10 min, the HB was dropped onto the specimen and kept at 55°C in a moist chamber for 2 h. The HB was then replaced with HB containing each probe and kept at 55°C in a moist chamber for 15–16 h. Then, the specimens were washed with washing buffer containing 50% formamide and 2 × SSC for 30 min at 65°C, followed by 2 × SSC for 20 min at 65°C and 0.2 × SSC twice for 20 min each at 65°C. Specimens were immersed in DIG buffer 1 (0.1 M Tris-HCl pH 7.5, 0.15 M NaCl, 0.1% Tween-20) for 5 min, the DIG buffer I was replaced with new DIG buffer I containing 1.5% blocking reagent and kept at room temperature for 60 min. DIG buffer I containing anti-DIG antibody conjugated with alkaline-phosphatase at 1 : 8000 dilution was dropped onto the specimens and kept at 4°C overnight. The specimens were washed with DIG buffer I twice for 15 min each and rinsed with DIG buffer III (0.1 M Tris-HCl pH 9.5, 0.1 M NaCl, 50 mM MgCl_2_, 0.1% Tween-20) for 3 min. DIG buffer III containing nitroblue tetrazolium (NBT)/5-bromo-4-chloro-3-indolyl phosphate (BCIP) at 1 : 50 dilution was dropped onto specimens and kept at room temperature in a moist black box-chamber until signal development. After washing with PBS, 4% PFA in 1 M PB was dropped onto the specimens and allowed to stand for 15 min. After washing with distilled water, Mount-Quick-Aqueous (Daido Sangyo Co., Ltd., Tokyo, Japan) was dropped onto specimens and the samples were sealed with commercially available nail polish. The specimens were observed using an Axiovert 135 microscope (Carl Zeiss Microscopy, München, Germany). Each slide contains three specimens, and at least three experimental replicates were examined to determine the presence or absence of signals.

## Results

### Transcriptome analysis

**Figure 1:**
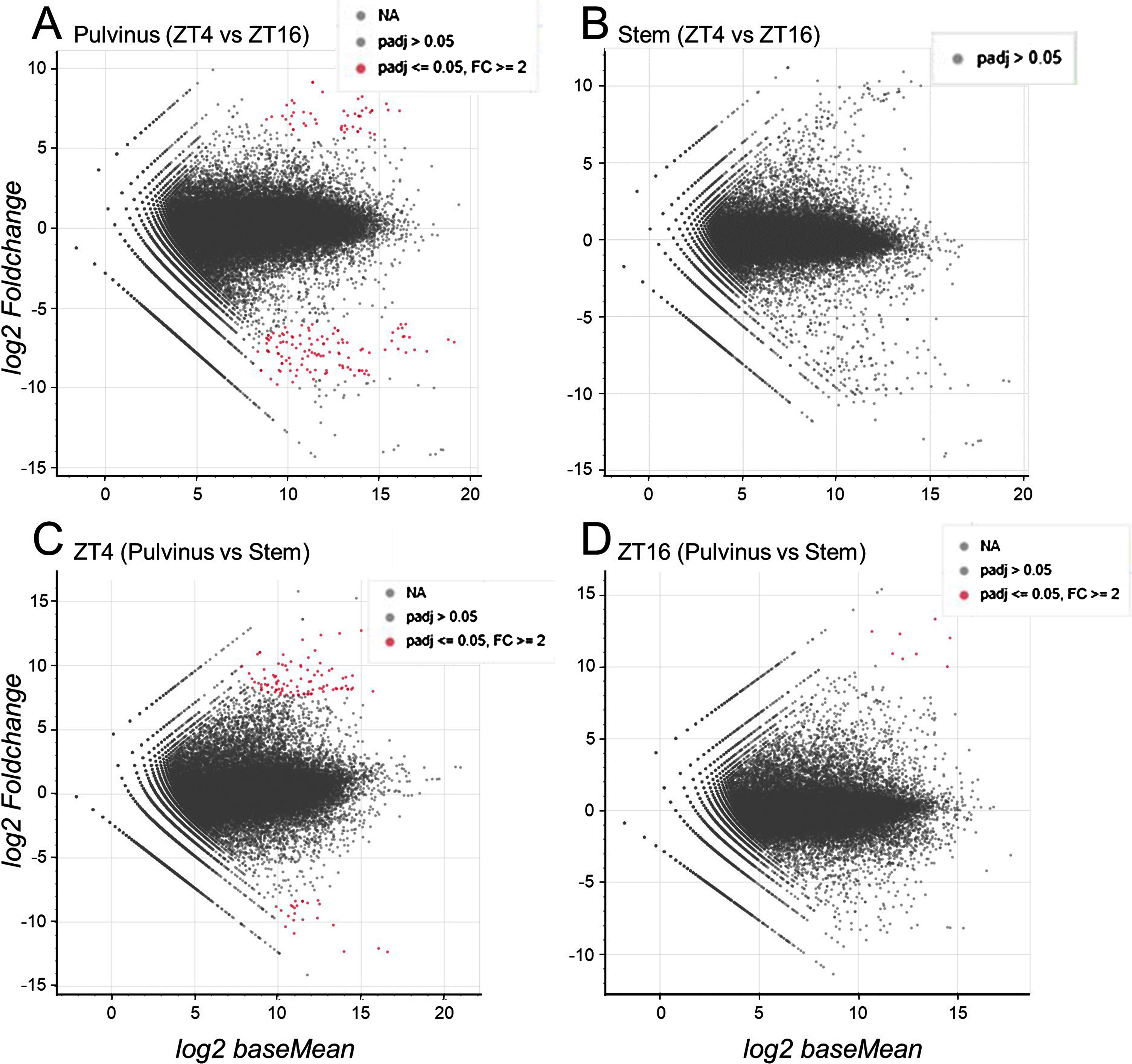
Figure 1. RNA seq analysis of differentially expressed genes in the pulvinus and stem of *L. japonicus*. MA plot analysis was used to visualize individual gene responses plotted as log_2_ fold change versus the log_2_ of the mean normalized counts of DEGs (differentially expressed genes) at different times (A and B; ZT4 vs. ZT16) or between samples (C and D; pulvinus vs. stem). Genes with significant differential expression (adjusted *p*-value, padj, less than 0.05, FC value greater than 2) are shown in red. The padj for the genes that do not pass the filter threshold are set to NA.

To analyze gene expression in the pulvinus, complementary DNA was prepared from pulvini and stems at 4 h after the start of the light period (ZT4) and at the start of the dark period (ZT16), and the sequences were analyzed by RNAseq. A total of 166 DEGs (49 up-regulated and 117 down-regulated) were found in the pulvinus between ZT4 and ZT16, whereas no DEGs were found in the stem (Figure 1A, B). Thus, many genes fluctuated in expression in the pulvinus between ZT4 and ZT16 compared to genes expressed in the stem. GO enrichment analysis shows that biological processes related to photosynthesis, aerobic respiration and mRNA processing are up-regulated at ZT4 (Supplementary Table S4), and cell wall organization is up-regulated at ZT16 (Supplementary Table S5). In addition, a comparison of genes expressed in the pulvinus and stem revealed that many genes were enriched in the pulvinus (23 DEGs at ZT4 and no DEGs at ZT16), with 23 DEGs classified mainly as tras-membrane ion transport (Supplementary Table S6). These results are consistent with previous reports (Oikawa et al. 2018; Wang et al. 2023), and led us to expect that the genes whose expression differed significantly between the two tissues included factors involved in the regulation of nyctinastic leaf movement.

To identify genes that are significantly up- or down-regulated in the pulvinus compared to the stem, we screened for genes based on the threshold of padj<0.05 and |Fold Change| >2 (Supplementary Tables S1 and S2). This study further investigates genes predominantly upregulated in the pulvinus. Among the screened genes, three genes, namely Sweet (sugar transporter, Lj4g3v2882250), ALMT (aluminum-activated malate transporter, Lj5g3v2261760), and ZnfC2H2 (C2H2 type zinc finger protein, Lj3g3v0488270), were of particular interest. These genes were identified as being of high rank in both screening periods. In addition to the above genes, we also examined the expression levels of the following genes by semiquantitative RT-PCR: i.e. genes listed in Supplementary Tables S1 and S2, such as INT (inositol transporter) and NRT (nitrate transporter), and genes that are dominantly expressed in the pulvinus at ZT4 (FLS1, flavonol synthase CYP82G1, cytochrome P450 family 82 protein, classified in cluster 8 of the heat map clustering (Supplementary Figure S3 and Table S9)) or ZT16 (CML41, calcium-binding protein; IAA19, auxin-responsive Aux/IAA gene; BAP2, BON1-associated protein 2-like, classified in cluster 7 or 13 (Supplementary Figure S3 and Table S10)). We also examined a gene that has been suggested to be expressed in the pulvinus (SKOR, Stelar K^+^ outward rectifier) (Moshelion et al. 2002).

**Figure 2:**
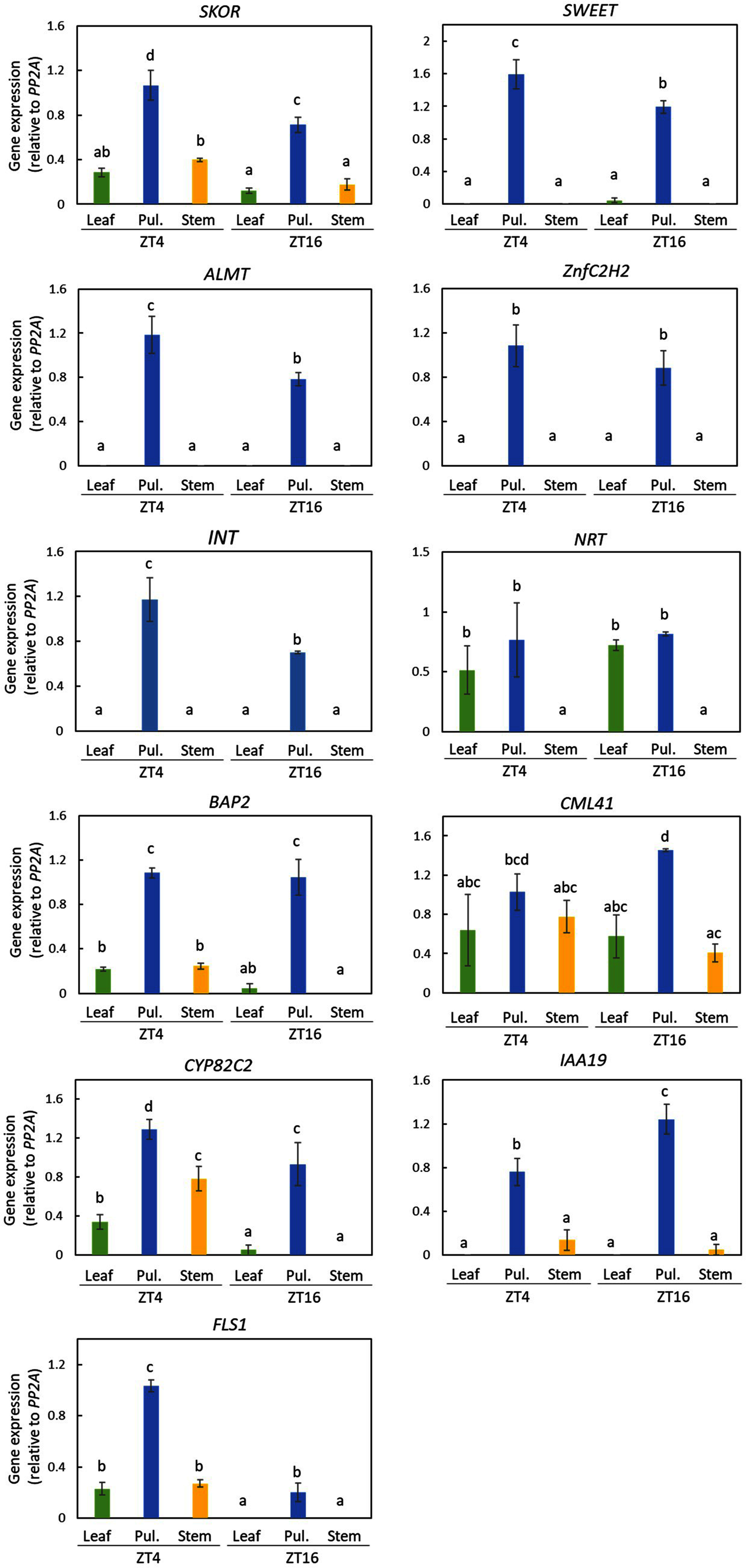
Figure 2. Tissue-specific gene expression analysis by semiquantitative RT-PCR. Total RNA was extracted from the leaves (Leaf), pulvinus (Pul.), and stem at zeitgeber time (ZT) 4 and 16. The genes listed in Table 1 were analyzed by RT-PCR. Relative transcript levels were determined using *PP2A* as a reference gene. Different letters represent significantly different means (ANOVA, Tukey’s post hoc test, *p*<0.05) between the samples. Each data point represents the mean±SEM from three individually harvested plants.

The results showed that the predominant expression of *Sweet*, *ALMT*, *ZnfC2H2*, *INT*, and *IAA19* was observed in the pulvinus. Although *SKOR* expression was detected in the pulvinus, its expression was also clearly detectable in other tissues. The expression of *NRT*, *BAP2*, *CML41*, and *CYP82C2* was identified not only in the pulvinus but also in other tissues (Figure 2 and Supplementary Figure S1). Interestingly, *FLS1* was expressed rather predominantly in the pulvinus, which was restricted to ZT4, consistent with the RNAseq data (Supplementary Table S7).

**Figure 3:**
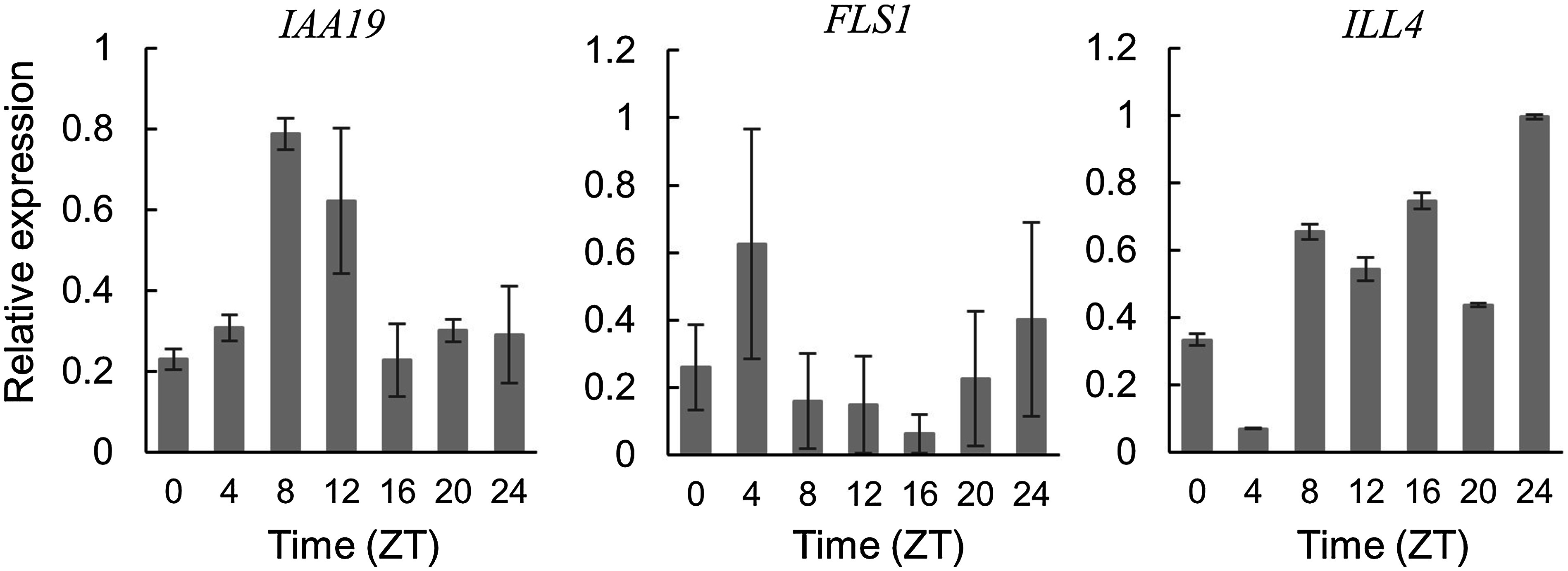
Figure 3. qRT-PCR analysis of auxin-related genes. Total RNA was extracted from the pulvinus at zeitgeber time (ZT) points every 4 h. Expression levels of *IAA19*, *FLS1*, and *ILL4* were normalized to *PP2A*. Each data point represents the mean±SE from three biological replicates.

In this study, we focused on auxin signals and found three related genes in the RNAseq data (Supplementary Table S7). IAA19 (similar to AUX22 of *Glycine max*) is predicted to form a complex with ARFs in the absence of auxin and repress the expression of down-stream genes. FLS (flavonol synthase) catalyzes the oxidation of dihydroflavonol to produce flavonols such as quercetin, myricetin, and kaempferol. Flavonols are known to control auxin transport (Kuhn et al. 2011). ILR1 (IAA-Leu-resistant1)/ILR1-like (ILL) is an amidohydrolase that cleaves IAA-amino acid conjugates to release active IAA (Bartel and Fink 1995), which was also found to be predominantly expressed in the pulvinus at ZT4 (Supplementary Table S7). The diurnal expression of these three genes was analyzed by qRT-PCR. A comparison of the relative expression to *PP2A* shows that *IAA19* is expressed during the day, while *FLS1* appears to be transiently expressed at dawn (Figure 3). These results suggest that FLS is involved in auxin influx into the motor cells of pulvinus and that the suppressor IAA19, whose degradation is enhanced by auxin at dawn, increases by feedback control at noon, affecting the subsequent decrease in osmotic pressure of the cell.

### In situ hybridization

To further examine the tissue distribution of auxin-related genes (*IAA19*, *FLS1*, and *ILL4*), the partial cDNA of each gene was subcloned into pGEM-T easy vectors, and the primary sequence was determined (Supplementary Figure S2). The obtained clones can be used as templates to generate probes for in situ hybridization.

**Figure 4:**
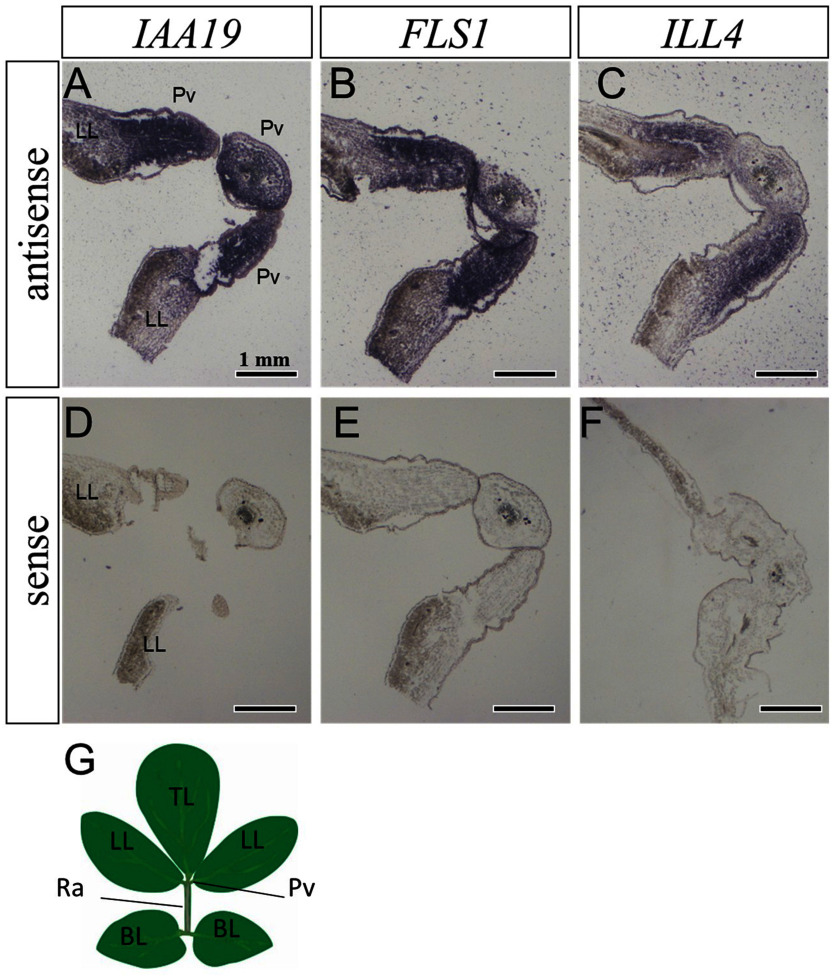
Figure 4. Tissue expression of the genes of interest. Sections prepared from plants at ZT4. Antisense and sense probes for in situ hybridization were used for the *IAA19* (A, D), *FLS1* (B, E), and *ILL4* genes (C, F). (G) Compound leaf illustration of *L. japonicus*. Pv, pulvinus; TL, terminal leaflet; LL, lateral leaflet; BL, basal leaflet; Ra, rachis.

Longitudinal sections were prepared from the pulvinus connecting to the rachis or leaflet and used for in situ hybridization. Signals were detected only with an antisense probe for each gene, not with a sense probe (Figure 4). As a result, for all genes, a strong signal was detected in the pulvinus and a weak signal was detected in the rachis.

## Discussion

In this study, the results of RNAseq analysis identified candidate genes that are predominantly expressed in the pulvinus. Extensive verification and analysis of the individual genes, including the creation of transgenic plants, will be needed in future work to confirm the involvement of these genes in the pulvinar nyctinastic movement. In advance of such efforts, we demonstrated the effectiveness of quantitative verification by PCR and relatively simple verification of tissue-specific expression by in situ hybridization.

We identified three genes (*Sweet*, *ALMT*, and *ZnfC2H2*) that are predominantly expressed in the pulvinus. Sweet and ALMT (Bauer et al. 2013; Sasaki et al. 2010) have been reported to contribute to stomatal opening/closing. We also identified the zinc finger-type transcription factor that is predominantly expressed in the pulvinus. Zinc finger protein ZmZFP3 of soybean has been reported to contribute to drought and salt responses (Zhang et al. 2016). However, there are no reports that these genes are present in the pulvinus or that they contribute to nyctinastic leaf movement. It has been reported that the *PLP* (petiole-like pulvinus)/*ELP* (elongated petiolule 1) gene of *Medicago truncatula* and the sleepless mutation of *L. japonicus* do not form normal pulvini, but rather cells with a petiole-like morphology. Consequently, PLP is purported to serve a pivotal function as a transcription factor that determines the morphology of the pulvinus (Chen et al. 2012; Kawaguchi 2003; Zhou et al. 2012). Zhou and colleagues also reported the comparison of gene expression in pulvinus and pulvinus-like petioles of *plp* mutant at day and night. They report that genes involved in secondary metabolism fluctuate in expression at night, and that genes involved in photosynthesis, lipid metabolism and protein synthesis may be involved in nyctinastic leaf movements during the day. Consistent with their findings, our results also showed that biological processes such as aerobic respiration and photosynthesis were activated at ZT 4 (Supplementary Table S4), suggesting that these elevated genes may be involved in the regulation of gene expression, such as ion transporters.

Interestingly, the transcriptome and proteome analyses of *M. truncatula* WT and *elp* mutant revealed that expressions of auxin pathway genes were altered in the pulvinus of the *elp* mutant, suggesting that ELP and auxin networks are necessary for normal pulvinus development in the plant (Bai et al. 2022). Although the genes involved in pulvinus morphogenesis are becoming increasingly clear, the network of genes responsible for their function has not yet been analyzed. The present study focused on auxin-related genes that are predominantly expressed in the pulvinus, as shown not only by semiquantitative RT-PCR but also by in situ hybridization techniques. Auxin is known to be involved in the promotion of plant development, as well as in nyctinastic leaf movement and pulvinar morphogenesis. The uptake of indole-3-acetic acid (IAA) through the cut pinnae of *M. pudica* showed that the leaves open in an IAA concentration-dependent manner even at night (Watanabe and Umrath 1983). Endogenous changes in auxin concentration contribute to the leaf opening in *Albizia julibrissin* (Satter et al. 1972). Further in vitro experiments reported that auxin can induce protoplast swelling in common beans (Iino et al. 2001). While the observation of auxin-regulated leaf movement has been reported (Wong et al. 2021), it is not yet clear which of the numerous auxin-related genes are involved in the leaf movement.

In this study, we screened for factors that may be involved in the nyctinastic leaf movement. By selecting genes that are highly expressed in the pulvinus rather than the stem, and whose expression changes during the diurnal cycle based on RNA-seq data, three auxin-related genes (*IAA19*, *FLS1*, and *ILL4*) were found. IAA19 is one of the ARFs whose expression is induced by auxin and forms a complex with ARFs in the absence of auxin to suppress gene expression. At least 29 genes of the Aux/IAA family have been identified in *Arabidopsis* and 17 different genes in the legume model plant *M. truncatula* (Remington et al. 2004; Shen et al. 2014). The search for ARFs that bind to these factors will help us to understand the molecular basis of auxin-signals in nyctinastic movement.

FLS is a catalytic enzyme for flavonol biosynthesis. It has been reported that flavonoids show maximum expression at dusk in response to drought stress (Ma et al. 2014). The expression of FLS1 at dawn may facilitate the transport of auxin in the motor cells of the pulvinus, increase the osmotic pressure of the cells via H^+^-ATPase, and accelerate the biodegradation of suppressor IAAs, followed by an increase in gene expression of ARF, including IAA19. ILL4 is a hydrolase of auxin precursors, converting the inactivate form of auxin to an active form in plant cells (Bartel and Fink 1995; LeClere et al. 2002). In higher plants, IAA is stored in a conjugated (inactive) form, and the active form of IAA is provided by hydrolysis by IRL1/ILL as needed during various developmental processes (Ludwig-Muller 2011). Thus, ILL4 may have a function in the growth and maintenance of the pulvinus.

Taken together, our report is a milestone that allows us to explore a new player that will be important in understanding the function and origin of pulvinus.

## Data Availability

The data supporting the results of this study are available from the corresponding author upon reasonable request.

## Description of Supplementary Files

Supplementary Table S1. Genes preferentially expressed in pulvinus in ZT4.

Supplementary Table S2. Genes preferentially expressed in pulvinus in ZT16.

Supplementary Table S3. Summary of Illumina RNA sequencing for samples.

Supplementary Table S4. GO enrichment analysis of DEGs upregulated in pulvinus at ZT4.

Supplementary Table S5. GO enrichment analysis of DEGs upregulated in pulvinus at ZT16.

Supplementary Table S6. GO enrichment analysis of DEGs expressed preferentially in pulvinus at ZT4.

Supplementary Table S7. FPKM values of auxin-related genes.

Supplementary Table S8. Primer list for cDNA cloning.

Supplementary Table S9. Gene list categorized in clusters 8 and 12.

Supplementary Table S10. Gene list categorized into clusters 7 and 13.

Supplementary Figure S1. Representative data of RT-PCR.

Supplementary Figure S2. Primary sequences of auxin-related genes.

Supplementary Figure S3. Hierarchically clustered heat map of the gene expression.
