# Supplementary material for: Expression analysis of genes enriched in the pulvinus of Lotus japonicus
Source: Plant Biotechnol (Tokyo). 2025 Mar 25;42(1):31–9. doi: 10.5511/plantbiotechnology.24.1030a (PMC12622902; doi:10.5511/plantbiotechnology.24.1030a)
Supplement: Supplementary Data [file plantbiotechnology-42-1-24.1030a-s001.pdf]

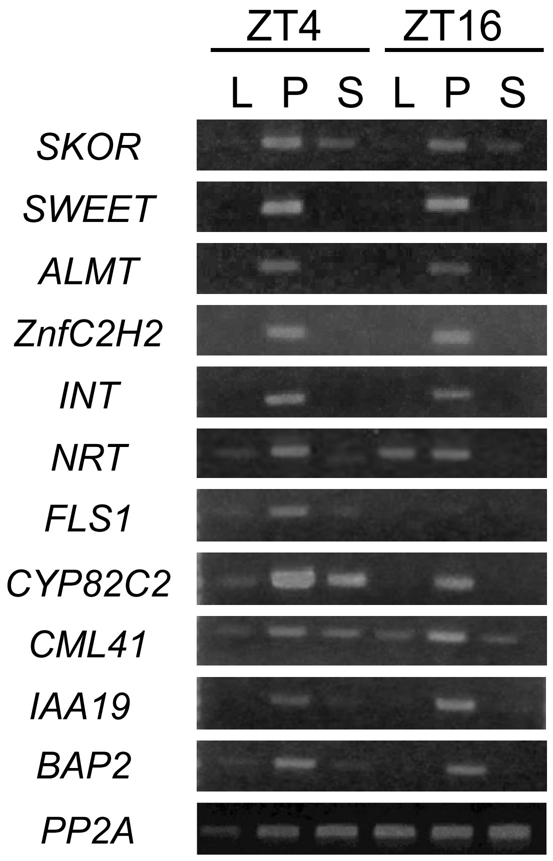

Supplementary Figure S1. Representative data of RT-PCR

Total RNA was extracted from the leaves (L), pulvinus (P), and stem (S) at zeitgeber time (ZT) 4 and 16. The genes listed in Table 1 were examined by RT-PCR.

>*IAA19*
cttgacctgtcattgttcccactACCTTCTTTTCCATTCACTTTGCATATATATATAACTAAACCC
TTTAATTTCTTCCATACTCAAAACCATTGATCTTTCAGCAAGCAAGAGAAAAAAGAA
GTTATTATTTGTGAAGTAATTTGTTGAGAAAATGGCGAAAGAAGGTCTTGGACTTG
AAATCACTGAGCTAAGGTTGGGTTTGCCTGGTGGGGAACGTATGAGTGACAAGAAT
GAGAAGAAGAGGGTGTTCTCAGAGATTGAAGGTGGCGGCGGGGATGAGAATAGCC
GCTCCGGTGAGCGGAGAGTGGAGAAGAAGAGTGAAGTGGTGGTGGGGTGGCCTC
CGGTGTGCTCATACCGGAAGAAGAACAGCGTGAATGAAGCTTCAAAAATGTATGTG
AAGGTTAGCATGGATGGGGCTCCTTTCTTGCGTAAAATTGACCTCAGCATGCATAA
GGGGTATTCTGATTTGGCTTTTGCTTTGGAGAAgctctttggttgctatggaatgg

>*FLS1*
atggagaaagtgagggtgcaagaCGTGGCTTCCCATTCCAACGATTCAATTCCCGCGGAGTTT
GTGAGATCAGAGACAGAGCAACCGGGCATCACGACTGTTAAGGGGACACAACTCG
AGGTTCCAGTCATTGATTTCAGCAACCCTGATGAGGACAAGGTCTTGAAAGAGGTT
GTGGAAGCAAGTTGTAAGTGGGGAATGTTTCAAATTGTGAACCATGAGATGCCGGT
TGAAGTTATAAGGGAACTTCAAGCTGTGGGGAAAACGTTCTTTGAGTTGCCTCAAG
AGGAAAAGGAGCAATATGCTAAACCTGCTGAATCTCAATCCATTGAAGGATATGGC
ACAAGGCTTCAGAAGGAAGTGGATGGGAAGAAAGGTTGGGTGGACCATTTGTTTC
ATAAGACATGGCCAACTTCTGATATTAACTACCGTTTCTGGCCTAAGAATCCTCCAT
CTTACAGAGAGGTTAATGAGGAATACAACAAGTACTTGCATGGGGTTGTAAACAAG
CTATTCAAAAAcctttcaatagggttaggccttg

>*ILL4*
gtttactataaatctgtgttgaaTTCACAATGACAAGAACACTGCAACTGCTACTGCATAAAGT
CTAGTGATTTTTGCTCCTCCACCATACCCACCCGCCTAAAAAATTCATAGAAGCCAT
GAATTTCTTCAAGTGGGTCAACTGGTTCATCATTTTCCTCTGCTTTGCTGCAACACA
GATCTTCTCAGATGAACACTCTGCAACCAAGTTTCTGGATTTTGCAAAGGAGCCTC
AGGTTTTTGATTGGATGGTGAATATCAGGAGGAAGGTTCATGAGAATCCAGAACTG
GGTTATGAGGAATTTGAGACTAGTAAGCTGATTAGAGCAGAATTGGATAAATTGGG
TATCACATATAAACATCCAGTTGCAGTTACAGGTGTTATTGGCTTCATAGGGACTGG
ACTTCCCCCTTTTGTTGCAATTAgggctgatatggatgctcttcct

Supplementary Figure S2. Primary sequences of auxin-related genes.

Partial cDNA sequences of IAA19, FLS1, and ILL4 were amplified by PCR and subcloned into pGEM-T easy vector. The clones obtained were used to synthesize probes for *in situ* hybridization. Lowercase letters indicate PCR primer sequences

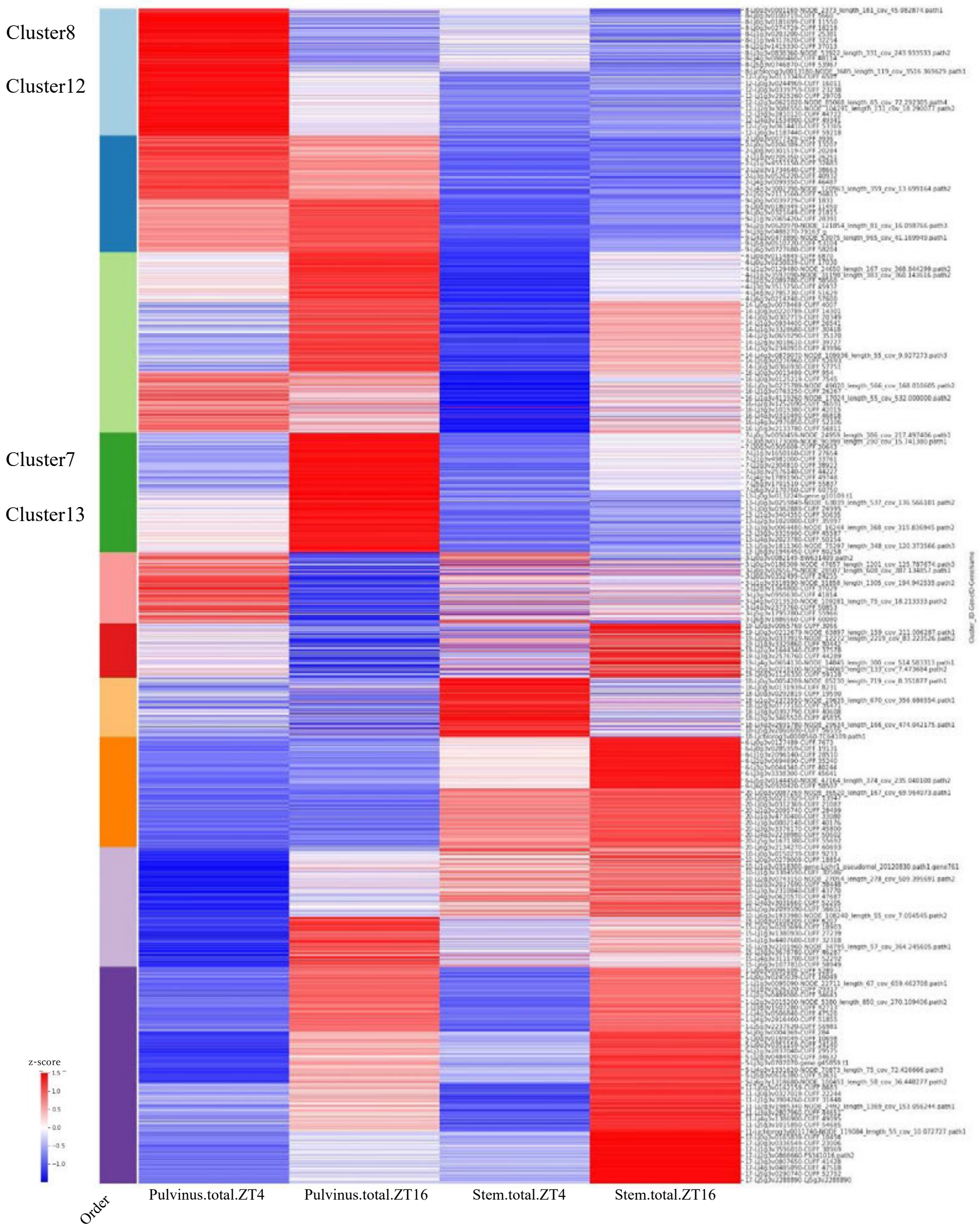

Supplementary Figure S3. Hierarchically clustered heat map of the gene expression.

A model-based clustering R package MBCluster.Seq was used for differential expression analysis. Hue represents the z-score of genes with the average FPKM ranging from 10 to 100. Cluster 8 and 12 are upregulated genes in pulvinus at ZT4. Cluster 7 and 13 are upregulated genes in pulvinus at ZT16. The genes are listed in Supplementary Table S8 and S9, respectively.
